# Identification of B-Cell Epitopes for Eliciting Neutralizing Antibodies against the SARS-CoV-2 Spike Protein through Bioinformatics and Monoclonal Antibody Targeting

**DOI:** 10.3390/ijms23084341

**Published:** 2022-04-14

**Authors:** Hui Xuan Lim, Malihe Masomian, Kanwal Khalid, Asqwin Uthaya Kumar, Paul A. MacAry, Chit Laa Poh

**Affiliations:** 1 Centre for Virus and Vaccine Research, School of Medical and Life Sciences, Sunway University, Bandar Sunway, Petaling Jaya 47500, Selangor, Malaysia; huixuanl@sunway.edu.my (H.X.L.); malihem@sunway.edu.my (M.M.); 19115914@imail.sunway.edu.my (K.K.); asqwin22@hotmail.com (A.U.K.); 2 Life Sciences Institute, National University of Singapore, Singapore 119077, Singapore; micpam@nus.edu.sg

**Keywords:** B-cell epitope, vaccine, SARS-CoV-2, spike protein

## Abstract

Severe acute respiratory syndrome coronavirus 2 (SARS-CoV-2) has caused a global public health crisis. Effective COVID-19 vaccines developed by Pfizer-BioNTech, Moderna, and Astra Zeneca have made significant impacts in controlling the COVID-19 burden, especially in reducing the transmission of SARS-CoV-2 and hospitalization incidences. In view of the emergence of new SARS-CoV-2 variants, vaccines developed against the Wuhan strain were less effective against the variants. Neutralizing antibodies produced by B cells are a critical component of adaptive immunity, particularly in neutralizing viruses by blocking virus attachment and entry into cells. Therefore, the identification of protective linear B-cell epitopes can guide epitope-based peptide designs. This study reviews the identification of SARS-CoV-2 B-cell epitopes within the spike, membrane and nucleocapsid proteins that can be incorporated as potent B-cell epitopes into peptide vaccine constructs. The bioinformatic approach offers a new in silico strategy for the mapping and identification of potential B-cell epitopes and, upon in vivo validation, would be useful for the rapid development of effective multi-epitope-based vaccines. Potent B-cell epitopes were identified from the analysis of three-dimensional structures of monoclonal antibodies in a complex with SARS-CoV-2 from literature mining. This review provides significant insights into the elicitation of potential neutralizing antibodies by potent B-cell epitopes, which could advance the development of multi-epitope peptide vaccines against SARS-CoV-2.

## 1. Introduction

Severe acute respiratory syndrome coronavirus 2 (SARS-CoV-2) first emerged in December 2019 in the Chinese city of Wuhan (Hubei Province), and it has led to a serious global health problem, causing a pandemic with over 452 million infections and high mortality, with more than 6.02 million deaths as of 11 March 2022.

The most common symptoms reported for SARS-CoV-2 infections are fever, dry cough, difficulty in breathing, and muscle pain, which may potentially worsen to pneumonia, renal failure, and death in severe cases [1,2]. Pneumonia was reported as the initial clinical symptom that indicated SARS-CoV-2 infection. Gastrointestinal symptoms were also observed. With a mean incubation period of five days, symptoms are observed in less than a week. In severe cases, dyspnoea and chest symptoms associated with pneumonia were reported in 75% of patients, as confirmed by computed tomography (CT) scans [3]. Severe symptoms in line with pneumonia were usually reported in the second or third week and were associated with reduced oxygen saturation, abnormal chest X-rays, alveolar exudates, and interlobular involvement, which demonstrated deterioration. Lymphopenia was reported with increased levels of inflammatory markers such as C-reactive protein and proinflammatory cytokines [4].

## 2. Genomic Structure of SARS-CoV-2

**Figure 1:**
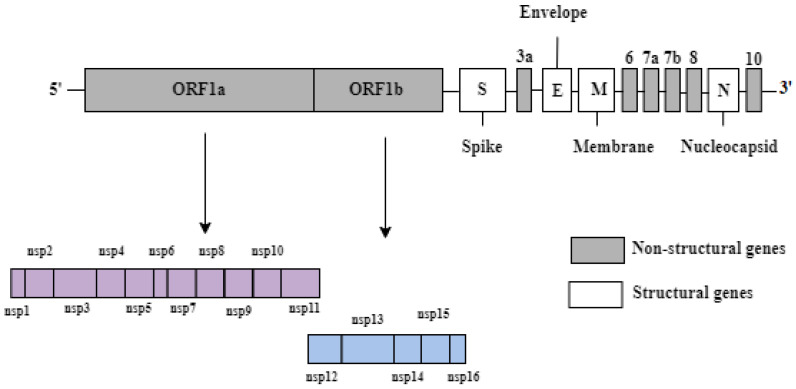
The genomic structure of SARS-CoV-2.

The SARS-CoV-2 genome consists of a single-stranded positive-sense RNA with a size of approximately 29.9 kB [5]. Two large open-reading frames (ORFs) comprising 70% of the genome, namely ORF1a and ORF1b, are located at the 5′ end (Figure 1). They are responsible for encoding 16 non-structural proteins, ranging from NSP1 to NSP16, which are involved in the formation of a replication–transcription complex (RTC). The RTC is associated with genome transcription and replication. The NSP genes have other diverse functions in terms of the proteins they encode, such as the cleavage of polypeptides and inhibition of the host immune response [6]. The other ORFs located at the 3′ end occupy 30% of the genome and are responsible for encoding four structural proteins, namely spike (S), envelope (E), membrane (M), and nucleocapsid (N) proteins (Figure 1). The S protein recognizes the angiotensin-converting enzyme 2 (ACE2) receptor, the M protein provides shape and structure to the viral particles, the E protein ensures proper virion assembly and release, and the N protein packages the RNA genome and enhances the pathogenicity by reducing interferon production. The 3′ UTR also encodes six accessory proteins labelled 3a, 6, 7a, 7b, 8, and 10 in Figure 1, but their functions are not fully known [7].

## 3. Viral Binding and Entry into Host Cell and the SARS-CoV-2 Lifecycle

An understanding of the functional characteristics of the S protein in the lifecycle of SARS-CoV-2 is essential, since the S protein mediates the binding of the virus and its entry into the host cells [8]. The S glycoprotein is a glycosylated type I membrane protein and consists of two subunits, S1 and S2. The S protein exists in a trimeric prefusion form, which is cleaved by a host furin protease into S1 and S2. The S1 subunit contains the NH-2 terminal domain (NTD) and the receptor-binding domain (RBD). The latter is a crucial structural component that is responsible for binding to the ACE2 cell receptor [9].

The S protein requires priming by host cell proteases such as endosomal cysteine protease cathepsin L and the serine proteases furin and TMPRSS2 in order to facilitate viral entry and membrane fusion. Cleavage of the S protein is known to occur at two sites. The first is the S1/S2 site by furin, which exposes the RBD in the “up” conformation and allows it to bind to the ACE2 receptor. The RBD promotes the binding of the enveloped virus to host cells by interacting with the ACE2 receptor expressed in the lower respiratory tract. After the RBD binds to ACE2, an additional cleavage of the S2 subunit occurs at a second specific site of the fusion peptide by the host serine protease TMPRSS2, which triggers the dissociation between S1 and S2. Cleavage at the S2 site produces the mature N-terminus of the fusion peptide, allowing the fusion of viral and host membrane, thus facilitating virus entry. When SARS-CoV-2 enters the host cell, it will release its RNA, and polyproteins will be produced following translation. The RNA genome of SARS-CoV-2 is replicated to yield genomic RNA, which is encapsidated by the viral structural proteins formed in the host cell. The newly assembled viral particles are released by exocytosis [2].

The spike glycoprotein is composed of two subunits, S1 and S2. It is an important antigenic determinant capable of inducing a protective immune response. The S1 subunit contains the RBD, which is the main target for SARS-CoV-2-neutralizing antibodies and convalescent serum titres against the RBD correlated well with neutralization titres [10]. The RBD in the SARS-CoV-2 spike protein is a crucial antigenic region, as it contains the interacting surface for ACE2 binding [11]. In addition, the RBD was reported to be immunodominant in the humoral response and accounted for 90% of neutralizing activities [12]. Therefore, most of the current SARS-CoV-2 vaccines were designed to target the S protein as an antigen to elicit humoral immune responses.

## 4. Current Status of SARS-CoV-2 Vaccine

The urgency of combating COVID-19 has fast-tracked vaccine development. RNA vaccines such as mRNA vaccines were rapidly manufactured, and they elicited strong humoral immune responses in clinical trials. The WHO reported a total of 140 vaccines candidates in clinical trials using various platforms by January 2022. The majority of the vaccine candidates are protein subunit vaccines (34%), RNA vaccines (17%), and non-replicating viral vectored vaccines (14%). Among the 10 vaccines approved for use by the World Health Organization (WHO), two were mRNA vaccines (BNT162b2 Pfizer and mRNA-1273 Moderna); three were non-replicating viral vector vaccines (Janssen Ad26.CoV2.S, Astrazeneca AZD1222, and Covishield); three were inactivated vaccines (CoronaVac, BBIBP-CorV, and Covaxin); and two were protein subunit vaccines (Novavax NVX-CoV2373 and COVOVAX). These vaccines have now been administered to millions of people globally, but the protective efficacies of COVID-19 vaccines have been reported to decline due to the emergence of new variants of concern (VOC). Pfizer’s BNT162b2 and Moderna’s mRNA-1273 vaccines were at least 10 times less effective against the B.1.351 (beta) variant. Ad26.COV2.S elicited 5.0- and 3.3-fold lower neutralizing antibody titres against the B.1.315 (beta) and P.1 (gamma) variants, respectively [2]. The efficacy of Astra Zeneca’s ChAdOx1 against the B.1.617.2 (delta) variant was 59.8% and only 22% against the B.1.351 (beta) variant [3,4]. More recently, the B.1.1.625 (Omicron) variant has already become the dominant variant of concern in many countries. The efficacy of Pfizer’s vaccine was reduced to 70% during the proxy Omicron period in South Africa [13]. Two doses of Pfizer vaccine were reported to elicit 41-fold less neutralizing antibodies against the Omicron variant [14]. Sera from vaccinees who received two doses of ChAdOx1-S and BNT162b2 were found to neutralize the Omicron variant to a much lesser extent when compared to the other variants (alpha, beta, or delta) [15]. The development of a vaccine for each major type of SARS-CoV-2 variant is impractical. A different strategy necessitates the search for highly conserved B-cell epitopes is a prerequisite for constructing an efficient multi-epitope peptide vaccine that can confer broad and long-term protection against the SARS-CoV-2 variants. This will halt the need to revaccinate with the current SARS-CoV-2 vaccines, which were developed based on the S antigen of the “Wuhan” strain, or for the vaccine manufacturers to continue making new vaccines to keep up with the emergence of new variants.

Since mRNA vaccine platforms are available, Pfizer and Moderna are more likely to develop Omicron-based vaccines. Pfizer and Moderna are currently developing vaccines based on the Omicron genome. Pfizer has initiated a clinical study to evaluate the safety, tolerability, and immune response of an Omicron-specific vaccine in healthy adults from 18 to 55 years of age [16], while the Omicron-specific vaccine candidate developed by Moderna (mRNA-1273.529) is undergoing evaluation in a Phase II clinical study [17].

The neutralization of Omicron variants in individuals receiving mRNA-1273 or BNT162b boosters were four to six-fold lower than the wild-type [18], but vaccinees are still protected from severe disease and hospitalizations. However, neutralization titres against the Omicron variant 6 months after the third (booster) dose of mRNA-1273 vaccine declined 6.3-fold from the peak titres assessed 1 month after the booster injection [19]. Although protection against Omicron could be provided by the third dose of the mRNA-1273 vaccine, the decrease in titres observed after 6 months might lead to the requirement for the use of a new Omicron-based vaccine to increase the duration of protection.

## 5. Approaches to Rational Design of Peptide Vaccines

New vaccine technologies based on subunit proteins or peptides require the identification of suitable antigens. Peptide-based vaccines are safer when compared to traditional vaccines (live attenuated and inactivated) due to minimal allergic and toxic properties [20]. The identification of peptide epitopes using phage display libraries, overlapping peptides that cover the whole length of the protein, or peptide arrays are costly and laborious. Recent advancements of bioinformatics approaches utilizing computational algorithms such as BepiPred, ABC pred, Discotope, and CBtope rely on amino acid sequences or 3D structures to predict B-cell epitopes [21]. The process of peptide vaccine development involves the identification of peptides specifying immunodominant epitopes according to the selected criteria, such as surface accessibility, high hydrophilicity, and antigenicity [22,23]. Multiple peptides were then joined with appropriate linkers and inserted into the expression vector pET-28a(+) so that the vaccine can be expressed in the bacterial system [24]. However, even though bioinformatics approaches will enable the predictions of potential antigenic epitopes, the immunogenicity of the epitopes has to be experimentally validated. Potent B-cell epitopes targeted by monoclonal antibodies are more likely to be conformational, which are difficult to incorporate into multi-epitope peptide-based vaccines. Short peptides comprising linear amino acids from discontinuous regions of the conformational epitopes can be incorporated into peptide-based vaccines. Hence, there is the need to identify B-cell epitopes from bioinformatics (prediction or validation) and monoclonal antibody targeting.

## 6. Identification of SARS-CoV-2 B-Cell Epitopes within S, M, and N Proteins from the Combination of Bioinformatics and In Vitro Neutralization Assays

Infection with SARS-CoV-2 initiates an immune response that leads to the production of binding antibodies. However, not all binding antibodies can block viral entry and replication. The subpopulation of binding antibodies known as neutralizing antibodies (nAbs) can neutralize the virus and thus prevent virus infection. They are elicited by neutralizing B-cell epitopes. The identification of epitopes that can induce robust B-cell responses is a prerequisite for designing epitope-based vaccines. Linear B-cell epitopes can be incorporated easily in the multi-epitope peptide vaccine to induce humoral responses. Besides linear epitopes, conformational B-cell epitopes can be identified by prediction methods such as ElliPro. In a recent study by Dong et al. (2020), three linear B-cell epitopes were selected for in silico cloning using the expression vector PET28a(+) [25]. However, further validations of the efficacy of the vaccines will be required. Conformational epitopes are more likely to be grated onto scaffolds such as virus-like particles [26] rather than being incorporated into multi-epitope peptide-based vaccines. Therefore, we focus on the search for immunogenic linear B-cell epitopes, which are highly conserved against SARS-CoV-2 for constructing an efficient multi-epitope peptide vaccine.

Poh et al. (2020) identified two immunodominant linear B-cell epitopes, S14P5 and S21P2, which were present on the SARS-CoV-2 S glycoprotein, by using pools of overlapping linear B-cell peptides spanning the entire S glycoprotein of SARS-CoV-2. Sera depleted of antibodies targeting either peptides S14P5 or S21P2 led to a >20% reduction in pseudotyped lentivirus neutralization, validating that antibodies targeting these two linear S epitopes are important for neutralizing SARS-CoV-2. Based on peptide arrays, Farrera-Soler et al. (2020) identified three immunodominant linear epitopes (S_655–672_, S_787–822_, and S_1147–1158_), which were recognized in >40% of COVID-19 patients. Two of these epitopes (S_655–672_ and S_787–822_) corresponded to key proteolytic sites on the spike proteins S1/S2 and S2, which have been shown to play a critical role in efficient viral entry [27]. Lu et al. (2021) predicted a total of 33 B-cell epitopes based on the 3D structure of the S, M, E, and N proteins, which were further elucidated by computational simulations on epitope surface accessibility. Six immunodominant linear B-cell epitopes were discovered: three were from the S protein; one from M; and two from N proteins (S_556–570_, S_675–689_, S_721–733_, M_183–197_, N_152–170_, and N_357–373_). However, the epitopes from the N protein are unlikely to be immunodominant B-cell epitopes, as the N protein is encapsidated within the virion and is inaccessible to antibody binding. The peptide S_556–570_ was also identified in a previous study as an immunodominant epitope that was able to elicit neutralizing antibodies [28]. As the S_556–570_ epitope is localized close to the RBD, it is plausible that antibodies binding to this region might sterically hinder the binding of SARS-CoV-2 to the ACE2 receptor, thereby abolishing the virus infection [29]. Among the 33 predicted epitopes, four peptides (S_92–106_, S_139–153_, S_439–454_, and S_455–469_) were able to elicit the production of neutralization antibodies against both D614 and G614 SARS-CoV-2 pseudoviruses with an inhibition rate of 40–50%. Epitope S_63–85_ induced the highest neutralizing effect on G614 SARS-CoV-2 pseudoviruses with an antibody titre of 1:80 [30].

**Figure 2:**
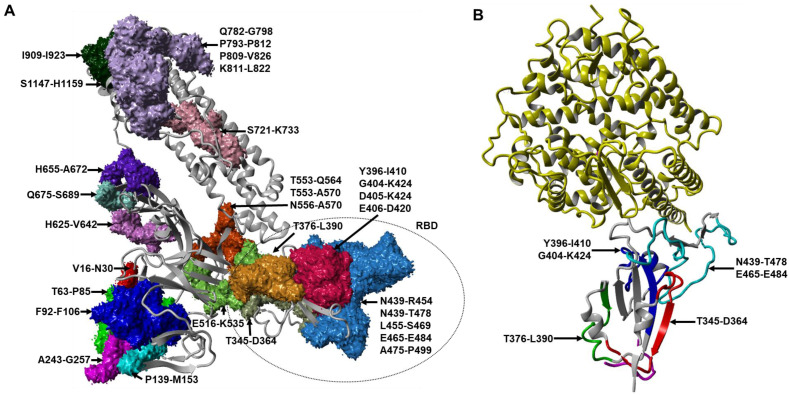
(**A**) The localization of B-cell epitopes mapped on the SARS-CoV-2 S monomer in closed conformation are represented by the amino residue number (PDB ID: 6ZB5/A). (**B**) Locations of B-cell epitopes targeted by monoclonal antibodies in the structure of ACE2 in complex with the SARS-CoV-2 RBD (PDB ID: 7DQA). ACE2 is shown in yellow, while the RBD is in grey colour.

It has been shown that 90% of neutralizing antibodies (nAbs) elicited against SARS-CoV-2 in COVID-19 patients were targeted at the RBD of the S glycoprotein [12]. Thus, profiling B-cell epitopes using sera from animals immunized with overlapping peptides spanning the RBD could reveal the molecular determinants of antigenicity. Three linear peptides specifying B-cell epitopes (R345, R405, and R465) were shown to elicit strong and specific IgG antibody responses from the SARS-CoV-2 S1 protein [31]. Another three B-cell epitopes present in the RBD, CoV2_S-10, CoV2_S-11, and CoV2_S-13, were identified by immunoinformatic predictions and confirmed by ELISA with sera from *Macaca fascicularis* vaccinated with a SARS-CoV-2 RBD subunit vaccine in the study published by Kanokporn Polyiam et al. (2021). In addition, the peptide S_404–424_ was also shown to elicit neutralizing antibodies in mice [32]. The epitope S_809–826_ (PSKPSKRSFIEDLLFNKV), which overlapped with the CoV2_S-17 epitope, has been demonstrated as a neutralizing epitope in humans [28]. Two epitopes that overlap with CoV2_S-20 (NNTVYDPLQPELDSFKEELDKYFKNHTSPDVDLGDISGI) have previously been characterized as immunodominant, as well as neutralizing [33,34]. The B-cell epitopes identified from the literature were mapped on the SARS-CoV-2 S monomer (Figure 2A), while the linear B-cell epitopes in the RBD targeted by monoclonal antibodies mined from the literature were mapped on the structure of the SARS-CoV-2 RBD and ACE2 complex (Figure 2B).

## 7. Monoclonal Antibodies against SARS-CoV-2 RBD Protein

Neutralizing antibody-mediated immunity protects an individual from viral infections by interfering with virus–host cell interactions required for viral attachment or entry. The majority of monoclonal antibodies isolated to date specifically target the RBD on the spike protein that allows SARS-CoV-2 to interact with the ACE2 receptor. Three monoclonal antibodies (15G9, 12C10, and 10D2) targeting the peptides R345, R405, and R465, respectively, were shown to inhibit the RBD–ACE2 interaction with an inhibition rate of 20–60%. This finding is consistent with a previous study where mAB 12C10 and mAb 10D2 exhibited 20–40% neutralization capacity [30]. Among the three mAbs, 12C10, which targeted the peptide R405, could strongly bind to both the SARS-CoV and SARS-CoV-2 S proteins, indicating that 12C10 is a cross-reactive antibody [31]. Antibodies targeting epitopes CoV2_S-10 and CoV2_S-11 were shown to inhibit RBD–ACE2 interactions [35]. The neutralizing potency of the antibody against epitope CoV2_S-10 was consistent with previous studies that reported an inhibition rate of 40% [30,31]. Monoclonal antibody B38, which could neutralize SARS-CoV-2, showed interactions with multiple residues in the RBD [36]. Murine antibodies induced by peptides S_406–420_ (EVRQIAPGQTGKIAD), S_439–454_ (NNLDSKVGGNYNYLYR), and S_455–469_ (LFRKSNLKPFERDIS), which corresponded to the epitopes present in CoV2_S-10 and CoV2_S11, were able to inhibit SARS-CoV-2 pseudovirus infections [30]. Wan et al. (2020) identified 11 potent neutralizing antibodies from 11 convalescent patients, and these also targeted three epitopes present in the RBD of the spike protein [37]. Amongst the three antibodies, antibody 414-1 showed the best neutralizing activity with an IC_50_ at 1.75 nM. Antibody 553-15 could substantially potentiate several other antibodies to have higher neutralizing abilities, while 515-5 showed cross-neutralizing activity towards the SARS-CoV pseudovirus. Two linear epitopes in the RBD were reported in the study of Makdasi et al. (2021). One of these epitopes spanning amino acids S_376–390_ (TFKCYGVSPTKLNDL) was targeted by antibodies 24 and 67, while the second epitope S_396–410_ (YADSFVIUGDEVRQI) was targeted by antibodies 69 and 90. The actual length of the second epitope could be further narrowed down to include only amino acids S_404–410_ (GDEVRQI) after visualizing the epitopes present on the crystal structure of the spike protein, as the first eight amino acids of the peptides were not exposed to the surface of the spike trimer [38]. The second epitope had seven overlapping amino acids with the epitopes S_404–424_ (GDEVRQIAPGQTGKIADYNYK) reported in a previous study [35]. Six representative epitopes covering the two hot spots (aa 525–685 and aa 770–829) across the S protein were selected from the screening of 211 peptides using peptide microarrays. Among the six selected epitopes, the antibodies against the three epitopes S_1–93_, S_1–105_, and S_2–78_ exhibited potent neutralizing activities with virus inhibitory efficiencies of 51%, 35%, and 35%, respectively. The antibody targeting S_1–93_ and S_553–564_ (TESNKKFLPFLPFQQ) showed the highest neutralizing capacity, which is consistent with the findings of a recent study [28].

Two potent neutralizing monoclonal antibodies, B38 and H4, which targeted different RBD sites, were capable of neutralizing live SARS-CoV-2 virus (IC_50_ = 0.177 μg/mL for B38 and 0.896 μg/mL for H4) [39]. The crystal structure of the RBD-B38 complex showed that most of the residues on the B38-binding epitope overlapped with the RBD–ACE2-binding interface, suggesting that B38 neutralized SARS-CoV-2 infection by functionally mimicking ACE2 to bind to RBD and blocked RBD–ACE2 binding. Importantly, a single dose of B38 or H4 (25 mg/kg) was demonstrated to reduce lung viral loads by 32.8% and 26% in mice, respectively, when compared to the untreated group [39].

CB6 was identified from the PBMCs of a COVID-19 convalescent patient by using the recombinant RBD of the SARS-CoV-2 S protein to screen memory B cells from PBMCs. Shi et al. (2020) showed that CB6 exhibited an effective neutralization of live SAS-CoV-2 infection of Vero-E6 cells with a neutralizing dose (ND50) of 0.036 ± 0.007 μg/mL. Structural studies revealed that B6 recognized an epitope that overlapped with angiotensin-converting enzyme 2 (ACE2)-binding sites in the SARS-CoV-2 receptor binding domain and thereby interfered with virus–receptor interactions by both steric hindrance and direct competition for interface residues. CB6 effectively reduced the viral loads and lessened infection-related lung damage in rhesus macaques [40].

Noy-Porat et al. (2020) isolated and characterized eight SARS-CoV-2-neutralizing monoclonal antibodies (nMAbs) that targeted four distinct epitopes on the RBD. These antibodies were selected from a phage display library constructed using peripheral circulatory lymphocytes collected from SARS-CoV-2 patients. Monoclonal antibodies MD45, MD67, MD62, and MD65 displayed the highest neutralizing potency, with a neutralization dosage (NT_50_) of 2.1, 1.9, 1.6, and 0.22 µg/mL, respectively. MD65 exhibited the highest neutralization capacity amongst all the monoclonal antibodies by completely inhibiting the binding of RBD to the ACE2 receptor [41].

A total of 25 mAbs were isolated from Epstein–Barr virus-immortalized memory B cells of a SARS-CoV-infected patient in 2003. S309 was the only mAb that had potent neutralizing activities against SARS-CoV-2 and SARS-CoV pseudoviruses, as well as the live SARS-CoV-2, by engaging the receptor-binding domain of the S glycoprotein. MAb S309 was shown to neutralize MLV-based SARS-CoV S-glycoprotein-pseudotyped viruses with IC_50_ of 120–180 ng/mL and displayed more potent neutralizing capability towards the live SARS-CoV-2 (2019n-CoV/USA_WA1/2020) with an IC_50_ of 79 ng/mL [42].

SARS-CoV-2 S protein-specific B cells were subsequently sorted for single-cell sequencing and mAb isolation. Among the 403 monoclonal antibodies isolated from three convalescent COVID-19 patients using a SARS-CoV-2-stabilized prefusion spike protein as the antigen, COVA1-18 and COVA2-15 were identified as unusually potent nMAbs targeting the RBD of the SARS-CoV-2 S protein. These mAbs showed neutralizing activities against the SARS-CoV-2 pseudoviruses in Huh7 liver cells with an IC_50_ value of 8 ng/mL and potently inhibited live SARS-CoV-2 infection in Vero-E6 cells with IC_50_ values of 7 and 9 ng/mL, respectively. Of the 19 mAbs that could inhibit SARS-CoV-2 pseudovirus infection, 14 were found to bind to the RBD [43].

A total of sixty-one neutralizing antibodies were isolated from the peripheral blood of five COVID-19 patients by sorting the S trimer-specific B cells, followed by single B cell receptor sequencing. Nineteen antibodies potently neutralized live SARS-CoV-2 in vitro. Nine NMAbs exhibited very high potency with 50% virus-inhibitory concentrations in the neutralizing range from 0.7 to 9 ng/mL, including four that were directed against the RBD, three directed against the N-terminal domain (NTD), and two directed against the nearby quaternary epitopes. The study reported that NMAb 2–15 is by far the most potent in the literature that targeted the RBD and could neutralize both pseudotyped and live SARS-CoV-2 virus in Vero-E6 cells with IC_50_ of 5 ng/mL and 0.7 ng/mL, respectively. A single dose of NMAb 2–15 (1.5 mg/kg) could effectively confer protection against SARS-CoV-2 infection in hamsters by 4-log reductions in virus titres [44].

Pinto et al. (2021) identified five mAbs from the memory B cells from three COVID-19 convalescent donors that targeted the conserved S2 stem helix region. Amongst the five mAbs, S2P6 had exceptionally broad cross-reactivity and neutralization against the SARS-CoV-2 variants (including Alpha, Beta, Gamma, and Kappa) and other beta-coronaviruses through the inhibition of membrane fusion. S2P6 was shown to neutralize the infection of live SARS-CoV-2 viruses to Vero-E6^+^ cells in the presence of protease TMPRSS2 with IC_50_ 1.67 µg/mL. S2P6 could neutralize SARS-CoV-2 pseudotyped with the S protein from several variants with IC_50_ ranging from 10 µg/mL to 100 µg/mL. Peptide mapping using linear 15-mers overlapping peptides revealed all five mAbs were binding to peptides S_1148–1156_ (FKEELDKYF) located in the S2 subunit. This peptide had nine overlapping amino acids with the B-cell epitopes reported in the studies of Farrera-Soler et al. (2020) and Li et al. (2020) [27,34]. Lastly, a single dose of mAb S2P6 (20 mg/kg) could effectively confer protection against the SARS-CoV-2 Wuhan strain and B.1.351 Beta variant infections in hamsters by 2-log and 1.5-log reductions in the lung viral RNA load, respectively [45]. The efficacies of the monoclonal antibodies recognizing the RBD of the S protein were listed in Table 1.

## 8. Conservancy of Linear B-Cell Epitopes against SARS-CoV-2 Variants

Previous studies have demonstrated that the RBD elicited the major pool of neutralizing antibodies, but it is too risky to focus only on the RBD, especially with the continuous emergence of novel SARS-CoV-2 variants throughout the world, which contain multiple mutations in the RBD protein. Thus, it would be interesting to assess the potential epitope conservancy towards different variants and to rationally design multiepitope peptide vaccines based on the best B-cell epitope combinations. A total of 200 unique spike (S) protein sequences for 11 SARS-CoV-2 variants of concern (VOC) and variants of interest (VOI) genes were retrieved from the NCBI (https://www.ncbi.nlm.nih.gov/ (accessed on 10 August 2021)) and GISAID databases (https://www.gisaid.org/ (accessed on 6 September 2021)). The nucleotide sequences obtained from GISAID were translated to amino acid sequences using the Expasy Translate Tool (https://web.expasy.org/translate/ (accessed on 21 October 2021)). The IEDB conservancy analysis tool (http://tools.iedb.org/conservancy/ (accessed on 12 January 2022)) [46] was used to compare the degree of conservation for peptides specifying B-cell epitopes by using protein sequences of the SARS-CoV-2 Wuhan strain and variants from worldwide isolates. The conservation score of the potent peptides specifying B-cell epitopes identified from cross-referencing of all the four SARS-CoV-2 variants are listed in Table 2. Amongst the five highly conserved (100%) peptides specifying B-cell epitopes that were reported in the literature, peptide S_345–364_ (TRFASVYAWNRKRISNCVAD) was reported to inhibit the RBD–ACE2 interaction with an inhibition rate of 60%. Thus, this peptide could improve and enhance the efficacy of the SARS-CoV-2 vaccine against the Wuhan strain and variants.

## 9. Conclusions

The development of an effective SARS-CoV-2 vaccine is challenging due to the emergence of SARS-CoV-2 variants. Producing a vaccine for each SARS-CoV-2 variant is impractical. A different strategy necessitates the search for highly conserved B-cell epitopes capable of eliciting neutralizing antibodies that can confer broad protection against the SARS-CoV-2 variants. This will halt the need to revaccinate with the current SARS-CoV-2 vaccines, which were developed based on the S-antigen of the “Wuhan” strain, or for the vaccine manufacturers to continually produce new vaccines to keep up with the emergence of new variants.

B-cell epitopes can be represented as either linear or conformational. Linear B-cell epitopes consist of linear sequences of amino acids that allow the binding of target-specific antibodies, whereas conformational epitopes are composed of discontinuous residues that are brought together in close proximity to form an antigenic site. Approximately 90% of the B-cell epitopes are conformational, and only a minority of B-cell epitopes are linear [26]. We aimed to review the immunogenic linear B-cell epitopes that are highly conserved, as well as conformational B-cell epitopes targeted by monoclonal antibodies such as CB6 or S309. There are a number of commonly used B-cell epitope prediction methods with high accuracy in cross-validation, such as CBTOPE and ElliPro [26]. The methods for conformational B-cell epitope prediction are challenging, as it generally requires the knowledge of the 3D structure of proteins. Conformational B-cell epitopes are unlikely to be incorporated into multi-epitope peptide-based vaccines that generally utilize linear peptides. However, linear amino acid sequences from discontinuous regions of the conformational epitopes can be extracted and incorporated in the peptide-based vaccine design [25]. Conformational epitopes are more likely to be grafted onto suitable scaffolds like virus-like particles (VLPs) that can mimic the native antigen [26].

To date, many SARS-CoV-2-neutralizing antibodies targeting RBD have been identified from convalescent patients and vaccinees. The efficacies of the neutralizing antibodies are often abrogated by RBD mutations in the spike protein. An unusually large number of mutations are found in the Omicron variants (B.1.1.529), consisting of more than 30 mutations in the spike protein. Indeed, studies have shown that Omicron would escape the majority of potent SARS-CoV-2-neutralizing antibodies that directly interfere with the binding of ACE2 reported from the literature [47]. However, neutralizing antibodies such as S309 and CR3022, which often exhibited broad sarbecovirus-neutralizing activities, were less affected by Omicron [47]. Therefore, the combination of highly conserved peptides with potent antigenicity in generating neutralizing antibodies would provide the rational basis for vaccine designs based on these B-cell epitopes.

**Table 1:** Efficacies of the monoclonal antibodies recognizing the RBD of the S protein.

mAb	Sources	Target	Efficacy	Protection	Reference
B38	Peripheral blood of SARS-CoV-2- infected patients	RBD	LV neutralization: IC_50_ = 0.177 µg/mL	Protection of mice: Lung viral loads reduced by 32.8% compared with PBS control.	[39]
H4	Peripheral blood of SARS-CoV-2- infected patients	RBD	LV neutralization: IC_50_ = 0.896 µg/mL	Protection of mice: Lung viral loads reduced by 26% compared with PBS control.	[39]
414-1	Peripheral blood of SARS-CoV-2- infected patients	RBD	LV neutralization IC_50_= 1.75 nM	N/A	[37]
MD65	Phage display library constructed using peripheral circulatory lymphocyte of SARS-CoV-2- infected patients	RBD	LV neutralization NT_50_= 0.22 µg/mL	N/A	[41]
COVA1–18	B cells of convalescent patients	RBD	PsV neutralization: IC_50_ = 0.008 µg/mL LV neutralization: IC_50_ = 0.007 µg/mL	N/A	[43]
COVA2-15	B cells of convalescent patients	RBD	PsV neutralization: IC_50_ = 0.008 µg/mL LV neutralization: IC_50_ = 0.009 µg/mL	N/A	[43]
2-15	Peripheral blood of COVID-19 patients	RBD	PsV neutralization: IC_50_ = 0.7 ng/mL LV neutralization: IC_50_ = 5 ng/mL	Protection of hamsters: Viral RNA copy numbers and infectious virus titers in lung tissues were reduced by 4 logs or more compared with the PBS control.	[44]
S309	Peripheral blood of SARS-infected patients	RBD	PsV neutralization: IC_50_ = 120~180 ng/mL	N/A	[42]
3F11	Humanized phage display library	RBD	PsV neutralization: IC_50_ = 3.8 ng/mL LV neutralization: IC_50_ = 436 ng/mL.	N/A	[48]
4A8	Peripheral blood of COVID-19 convalescent patients	NTD (in S1)	PsV neutralization: EC_50_ = 49 μg/mL LV neutralization: EC_50_ = 0.61 μg/mL	N/A	[49]
CR3022	Gene cloning; Protein expression	RBD	LV neutralization: IC_50_ = ~0.114 μg/mL	N/A	
CB6	B cells of convalescent patients	RBD	PsV neutralization: ND_50_ = 0.036 µg/mL LV neutralization: ND_50_ = 0.036 µg/mL	Protection of rhesus macaques: 50 mg/kg	[40]
S2P6	Memory B cells of SARS-CoV-2 patients	S2	LV neutralization: IC_50_ = 1.67 µg/mL PsV D614G: IC_50_~10 µg/mL PsV P.1: IC_50_~10 µg/mL PsV B.1.1.7: IC_50_~100 µg/mL PsV B.1.351: IC_50_~100 µg/mL PsV 1.1.617: IC_50_~20 µg/mL	Protection of hamsters: Viral RNA copy numbers in lung tissues were reduced by 2 logs and 1.5 logs against SARS-CoV-2 Wuhan strain and B.1.351 Beta strain.	[45]

LV: live viruses; PsV: SARS-CoV-2 pseudoviruses; N/A: data not available.

**Table 2:** Peptides specifying B-cell epitopes identified in SARS-CoV-2.

Protein	MonoclonalAntibody	Peptide ID	Start-End (aa)	Sequences of B-Cell Epitopes	Methods	Host	Inhibition	Conservancy (%)	Reference
S1 S2	N/A	S14P5 S21P2	553–570 809–826	TESNKKFLPFQQFGRDIA PSKPSKRSFIEDLLFNKV	Overlapping peptide library	COVID-19 sera	>20% of pseudoviruses >20% of pseudoviruses	84 100	[28]
S1 S2 S2	N/A	N/A	655–672 782–798/811–822 1147–1158	HVNNSYECDIPIGAGICA QIYKTPPIKDFG/KPSKRSFIEDLL SFKEELDKYFKN	Peptide array	COVID-19 plasma	N/A	93.50 98/100 100	[27]
S1/S2 S2	N/A	N/A	675–689 721–733	QTQTNSPRRARSVAS SVTTEILPVSMTK	Epitope predictions based on 3D protein structure, epitope surface accessibility	COVID-19 sera, BALB/c mice	~50% of G614 pseudoviruses No inhibition of D614 pseudoviruses	60.5 98.5	[30]
S1 S1 RBD RBD S1 S2 S2	N/A	N/A	16–30 243–257 406–420 475–499 556–570 793–812(N) 909–923	VNLTTRTQLPPAYTN ALHRSYLTPGDSSSG EVRQIAPGQTGKIAD AGSTPCNGVEGFNCYFPLQSYGFQP NKKFLPFQQFGRDIA PIKDFGGFN(GlcNAc)FSQILPDPSKP IGVTQNVLYENQKLI			20–40% inhibition of D614 pseudoviruses	84 85.50 88 53.50 84 99 99.50	
S1 S1 RBD RBD	N/A	N/A	92–106 139–153 439–454 455–469	FASTEKSNIIRGWIF PFLGVYYHKNNKSWM NNLDSKVGGNYNYLYR LFRKSNLKPFERDIS			40–50% inhibition of D614, G614 pseudoviruses	89.50 62 76.50 100	
S1	N/A	N/A	63–85	TWFHAIHVSGTNGTKRFDNPVLP			>80% inhibition of G614 pseudoviruses	65.50	
RBD RBD RBD	15G9 12C10 10D2	R345 R405 R465	345–364 405–424 465–484	TRFASVYAWNRKRISNCVAD DEVRQIAPGQTGKIADYNYK ERDISTEIYQAGSTPCNGVE	Overlapping peptides covering RBD	Swine and mice	60% of RBD/ACE2 interaction 40% of RBD/ACE2 interaction 20% of RBD/ACE2 interaction	100 88 57	[31]
RBD RBD RBD	N/A	CoV2_S-10 CoV2_S-11 CoV2_S-13	404–424 439–478 516–535	GDEVRQIAPGQTGKIADYNYK NNLDSKVGGNYNYLYRLFRKSNLKPFERDISTEIYQAGST ELLHAPATVCGPKKSTNLVK	Immunoinformatic prediction (Bepipred-2.0)	Cynomolgus macaques	N/A	88 74 98.50	[35]
S1 S1 S2	N/A	S1-93 S1-105 S2-78	553–564 625–642 1148–1159	TESNKKFLPFQQ HADQLTPTWRVYSTGSNV FKEELDKYFKNH	Peptide microarray	COVID-19 sera	51% of pseudoviruses 35% of pseudoviruses 35% of pseudoviruses	99 99.5 100	[34]
RBD RBD	Ab 24 & 67 Ab 69 & 90	N/A	376–390 396–410	TFKCYGVSPTKLNDL YADSFVIRGDEVRQI	Overlapping peptides covering S protein	Rabbit sera	N/A	86.67 53.33	[38]
**S2**	S2P6	N/A	1148–1156	KEELDKYF	X-ray crystallography and Cryo-EM	COVID-19 sera	>90% inhibition of live viruses	100	[45]

N/A: data not available; Peptide ID: identity of peptides.

## Data Availability

Not applicable.
